# Genome-wide identification of the *SPL* gene family in Tartary Buckwheat (*Fagopyrum tataricum*) and expression analysis during fruit development stages

**DOI:** 10.1186/s12870-019-1916-6

**Published:** 2019-07-08

**Authors:** Moyang Liu, Wenjun Sun, Zhaotang Ma, Li Huang, Qi Wu, Zizhong Tang, Tongliang Bu, Chenglei Li, Hui Chen

**Affiliations:** 1 College of Life Science, Sichuan Agricultural University, Ya’an, China; 2 School of Agriculture and Biolog, Shanghai Jiao Tong University, Shanghai, China

**Keywords:** Tartary buckwheat, *FtSPL*, Genome, Fruit development, Expression patterns

## Abstract

**Background:**

SPL (SQUAMOSA promoter binding protein-like) is a class of plant-specific transcription factors that play important roles in many growth and developmental processes, including shoot and inflorescence branching, embryonic development, signal transduction, leaf initiation, phase transition, and flower and fruit development. The SPL gene family has been identified and characterized in many species but has not been well studied in tartary buckwheat, which is an important edible and medicinal crop.

**Results:**

In this study, 24 *Fagopyrum tataricum SPL* (*FtSPL*) genes were identified and renamed according to the chromosomal distribution of the *FtSPL* genes. According to the amino acid sequence of the SBP domain and gene structure, the *SPL* genes were divided into eight groups (group I to group VII) by phylogenetic tree analysis. A total of 10 motifs were detected in the tartary buckwheat *SPL* genes. The expression patterns of *23 SPL* genes in different tissues and fruits at different developmental stages (green fruit stage, discoloration stage and initial maturity stage) were determined by quantitative real-time polymerase chain reaction (qRT-PCR).

**Conclusions:**

The tartary buckwheat genome contained *24 SPL* genes, and most of the genes were expressed in different tissues. qRT-PCR showed that *FtSPLs* played important roles in the growth and development of tartary buckwheat, and genes that might regulate flower and fruit development were preliminarily identified. This work provides a comprehensive understanding of the SBP-box gene family in tartary buckwheat and lays a significant foundation for further studies on the functional characteristics of *FtSPL* genes and improvement of tartary buckwheat crops.

**Electronic supplementary material:**

The online version of this article (10.1186/s12870-019-1916-6) contains supplementary material, which is available to authorized users.

## Background

Tartary buckwheat originated in Southwest China and is currently grown in Southwest China and the Himalayan region, as well as in Japan, Canada and other countries. As a widely cultivated medicinal and edible crop, tartary buckwheat is rich in complete proteins with a well-balanced composition of essential amino acids and is rich in beneficial soluble fiber and phytochemicals [1–4]. Tartary buckwheat fruits are rich in rutin, which has been reported to prevent liver damage [5]. The development and ripening of tartary buckwheat fruit are important stages of its growth and development. Transcription factors (TFs) play an indispensable role in regulating the growth and development of plants. At present, the auxin response factor gene family has been identified in tartary buckwheat, and the potential role of *Fagopyrum tataricum ARF2* (*FtARF2*) on the fruit size of tartary buckwheat has been studied [6, 7]. Because the SQUAMOSA promoter binding protein (SBP)-like (*SPL*) gene regulates inflorescence branching and grain development, it is important to explore the potential functions of *FtSPL* genes to understand the regulation of the growth and development of tartary buckwheat.

The SBP-box is an important transcription factor family that regulates growth and development in plants. It was first isolated from the cDNA library of *Antirrhinum majus* inflorescences and was named for its ability to recognize and bind to the promoter of SQUAMOSA (SQ-UA) [8, 9]. SPL is a general term for a class of transcription factors similar to SBP-box. The *SPL* genes encode a highly conserved SBP domain containing approximately 76 amino acid residues, including two tandem zinc fingers (Cys-Cys-His-Cys and Cys-Cys-Cys-His), and possess a nuclear localization signal (NLS) at the C-terminus [10, 11]. Studies have shown that SPL TFs exist widely in plants and play an important role in many aspects of plant growth and development, including regulating flower formation and late flower development, controlling plant transformation from vegetative growth to reproductive growth, regulating leaf morphogenesis, and responding to environmental signals [12–15].

With the release of plant genome data, the genome-wide identification and analysis of the whole SPL gene family have been carried out in many plants, such as *Arabidopsis thaliana* (*A. thaliana*) [16, 17], *Solanum lycopersicum* (*S. lycopersicum*) [18], *Glycine max* (*G. max*) [19], *Vitis vinifera* (*V. vinifera*) [20], *Malus domestica* Borkh (*M. domestica*) [21], *Ricinus communis* L (*R. communis* L) [22], *Zea mays* L (*Z. mays*) [23], *Capsicum annuum* L (*C. annuum*) [24], and *Salvia miltiorrhiza (S. miltiorrhiza)* [25]. In *A. thaliana*, the 16 *A. thaliana SPL* (*AtSPL*) genes were divided into 8 groups according to the amino acid sequence of the SBP domain: *AtSPL7* (I group), *AtSPL1/12/14/16* (II group), *AtSPL8* (III group), *AtSPL6* (IV group), *AtSPL2/10/11* (V group), *AtSPL3/4/5* (VI group), *AtSPL13* (VII group), and *AtSPL9/15* (VII group). The functions of these *SPL* genes in *A. thaliana* have also been identified, and they play an important role in leaf, flower and shoot development [15, 26, 27]. At present, many *SPL* genes have been isolated and identified in other plants, but studies on the SPL proteins in tartary buckwheat have not been conducted, and the functions of these proteins are not clear.

In this study, we comprehensively analyzed the gene structures, motif compositions, chromosomal locations, and gene duplications of 24 *SPL* genes on the basis of the recently completed genome sequence of tartary buckwheat and compared the evolutionary relationship of tartary buckwheat with *A. thaliana*, *Beta vulgaris* (*B. vulgaris*), *G. max, S. lycopersicum, V. vinifera,* and *Oryza sativa* (*O. sativa*)*.* Compared with the identification of SPL family in other plants, this study not only analyzed the grouping, gene structure, chromosome localization, tandem and fragment replication of FtSPL gene family, but also analyzed the evolutionary relationship of *FtSPL* genes in multiple species, including multiple species phylogenetic tree, motif composition and collinearity analysis among them. In addition, we studied the spatial expression of *SPL* genes in different tissues and their expression patterns during tartary buckwheat fruit development. Through global expression analysis, the roles of specific *SPL* gene family members in different biological processes of tartary buckwheat were determined. This study comprehensively analyzed SPL gene families in tartary buckwheat, not only providing valuable information for screening important *SPL* genes in tartary buckwheat growth and development, but also providing methods for mining SPL families in other plants.

## Results

### Identification of the *FtSPL* genes in tartary buckwheat

In this study, we extracted the *SPL* genes from the tartary buckwheat genome by the two BLASTp methods, and we identified 24 *SPL* genes from tartary buckwheat after removing the redundant sequences. We renamed the *SPL* genes based on their location on the chromosomes of tartary buckwheat (Additional file 2 Table S1). From the location information of the *SPL* genes, we saw that all *SPL* genes were located in the nucleus (Additional file 2 Table S1). The predicted SPL proteins of tartary buckwheat varied greatly in length and molecular weight (MV). The tartary buckwheat *SPL* genes encoded proteins ranging from 100 (FtPinG0006337300.01) to 1004 (FtPinG0000377600.01) amino acids (aa) in length and from 11.7 (FtPinG0006337300.01) to 110.77 (FtPinG0000377600.01) kDa in MV. The isoelectric points (PIs) of the proteins ranged from 5.69 (FtPinG0000377600.01) to 9.75 (FtPinG0001287900.01) (Additional file 2 Table S1).

### Multiple sequence alignment, phylogenetic analysis, and classification of the *FtSPL* genes

Multiple sequence alignment with the SPL full-length protein sequence showed that the SBP domain was basically conserved in specific positions, such as the CQQC sequence (235–238 amino acid), SCR sequence (253–256 amino acid) and RRR sequence (264–267 amino acid). Almost all sequences contained two zinc finger-like structures, Zn-1, Zn-2 and a highly conserved nuclear localization signal (NLS) (Additional file 1 Figure S1).

**Fig. 1:**
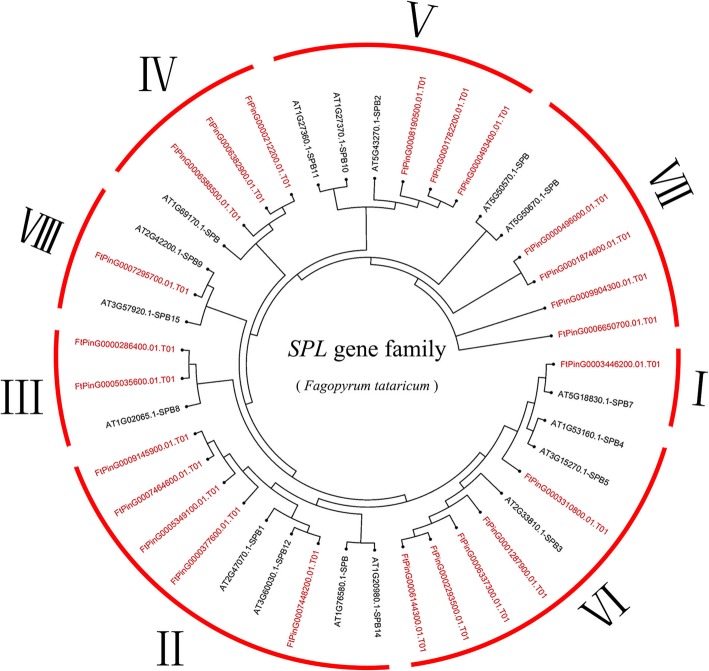
Unrooted phylogenetic tree representing the relationships among 24 *SPL* genes of tartary buckwheat and Arabidopsis. The genes in tartary buckwheat are marked in red, while those in Arabidopsis are marked in black.

To investigate the phylogenetic relationships of the *SPL* genes in tartary buckwheat, 24 tartary buckwheat *SPL* genes and 17 *A. thaliana SPL* genes were used to construct a neighbor-joining (N-J) phylogenetic tree using MEGA 6.0 software. From the phylogenetic tree, we saw that the *FtSPL* genes were divided into eight subgroups (I to VII). There was only one member from tartary buckwheat in group I and group VII, five members from tartary buckwheat in group II and group VI, three members from tartary buckwheat in group IV and group V, two members from tartary buckwheat in group III, and Group VII had four members from tartary buckwheat (Fig. 1).

### Gene structure and motif composition of the *FtSPL* gene family

**Fig. 2:**
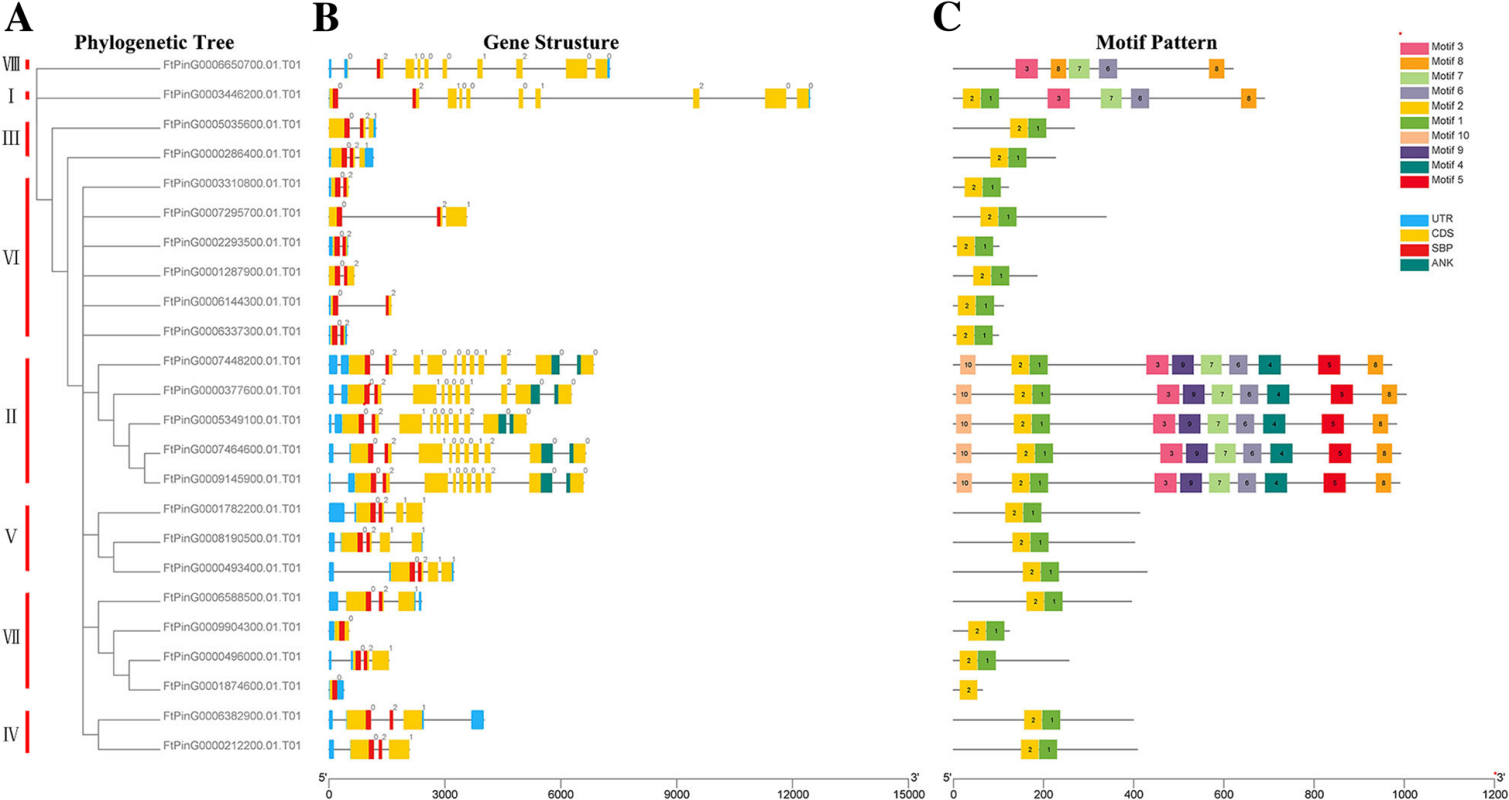
Phylogenetic relationships, gene structures and architectures of the conserved protein motifs of the *SPL* genes from tartary buckwheat. **a** The phylogenetic tree was constructed based on the full-length sequences of tartary buckwheat SPL proteins using Geneious R11 software. **b** Exon-intron structures of tartary buckwheat *SPL* genes. Yellow boxes indicate untranslated 5′- and 3′-regions; blue boxes indicate exons; and black lines indicate introns. The SBP domain is highlighted by a red box. The number indicates the phases of the corresponding introns. **c** The motif compositions of the tartary buckwheat SPL proteins. The motifs, numbered 1–10, are displayed in different colored boxes. The sequence information for each motif is provided in Table S2. The protein length can be estimated using the scale at the bottom.

When a phylogenetic tree based on the full-length predicted FtSPL protein sequences was constructed, the structural diversity of the tartary buckwheat *SPL* genes was examined, and these proteins were also roughly divided into eight subgroups (Fig. 2). The diversity of the genetic structure may be a mechanism to promote the evolution of multigene families. We compared the number and location of the exons and introns in the *SPL* gene sequences of tartary buckwheat and further explored the structural diversity of the *FtSPL* genes. From Fig. 2b, we saw that the genes in group II contained the largest number of exons, of which *FtPinG0007448200.01* contained 11 exons, while other genes contained 10 exons. The *FtSPL* genes in group VI all had two exons, which was the lowest number of exons among these subgroups. In general, the number of exons within the same subgroup was similar, although the locations of the exons were different.

Further analysis showed that there was a conserved SBP domain in the N-terminus of all FtSPL proteins and a conserved ANK domain only in the C-terminus of members of group II (FtPinG0007448200.01/377600.01/5349100.01/7464600.01/9145900.01) (Fig. 2b). We used MEME software to detect the motif compositions of the entire sequences, including the conserved domains in all FtSPL proteins, to explore the diversity of the motifs in each protein (Fig. 2c). A total of 10 different motifs were detected in the tartary buckwheat *FtSPL* genes and named motif 1 to motif 10. The length and sequences of the 10 motifs are listed in Table S3. The number of conserved motifs in each FtSPL protein ranged from 1 to 10. All FtSPL proteins except FtPinG0006850700.01 and FtPinG0001874600.01 contained motif 1 and motif 2, while those in group II contained all the motifs. Twelve FtSPL proteins only had motif 1 and motif 2, and FtPinG0001874600.01 only contained motif 2. In short, members belonging to the same subgroup had similar a gene structure and motif composition and tended to gather together in the phylogenetic trees.

### Chromosomal distribution, gene duplication and synteny analysis of the *FtSPL* genes

**Fig. 3:**
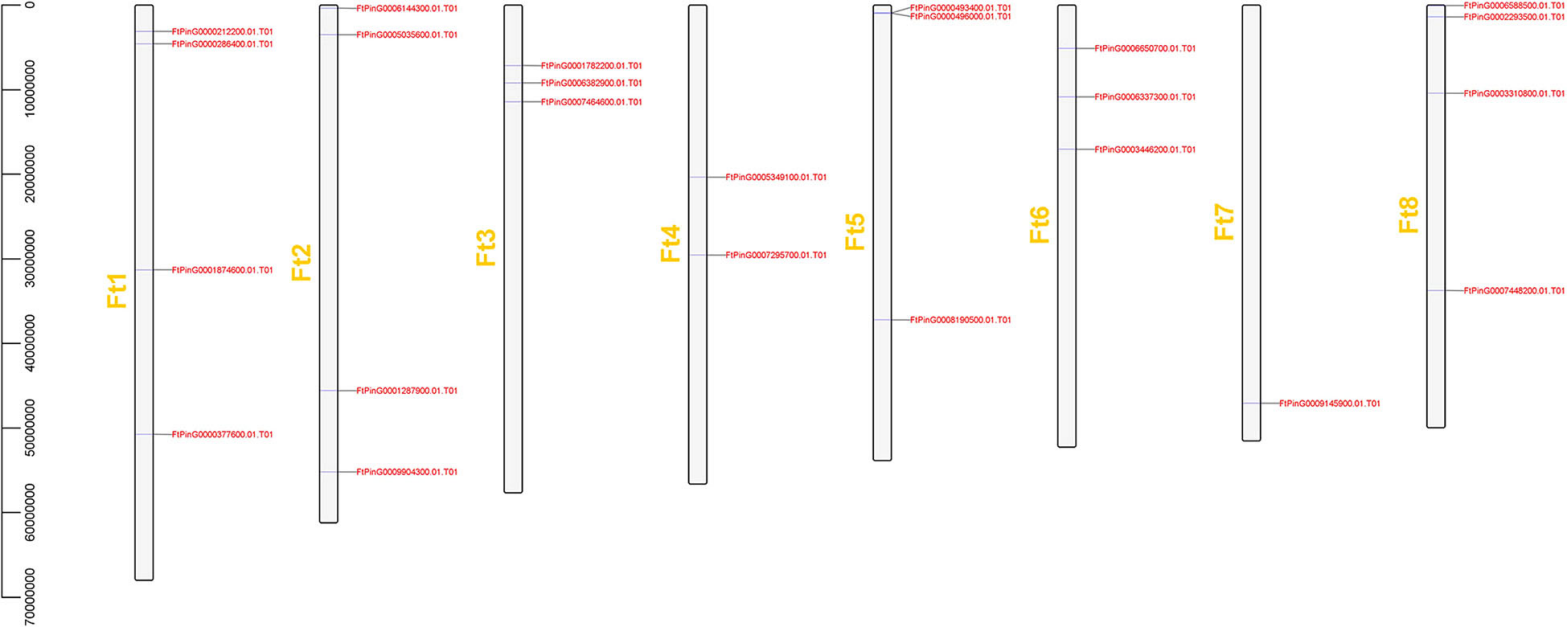
Schematic representations of the chromosomal distribution of the tartary buckwheat *SPL* genes. The chromosome number is indicated to the left of each chromosome.

The chromosome mapping of the *FtSPL* genes was performed using the latest tartary buckwheat genome database. A total of 24 *SPL* genes were unevenly distributed in eight tartary buckwheat linkage groups (LGs) (Fig. 3). There was only one gene (*FtPinG0009145900.01*) in LG 7. There were four genes in LG1, LG2 and LG8, which was the largest number of genes.

**Fig. 4:**
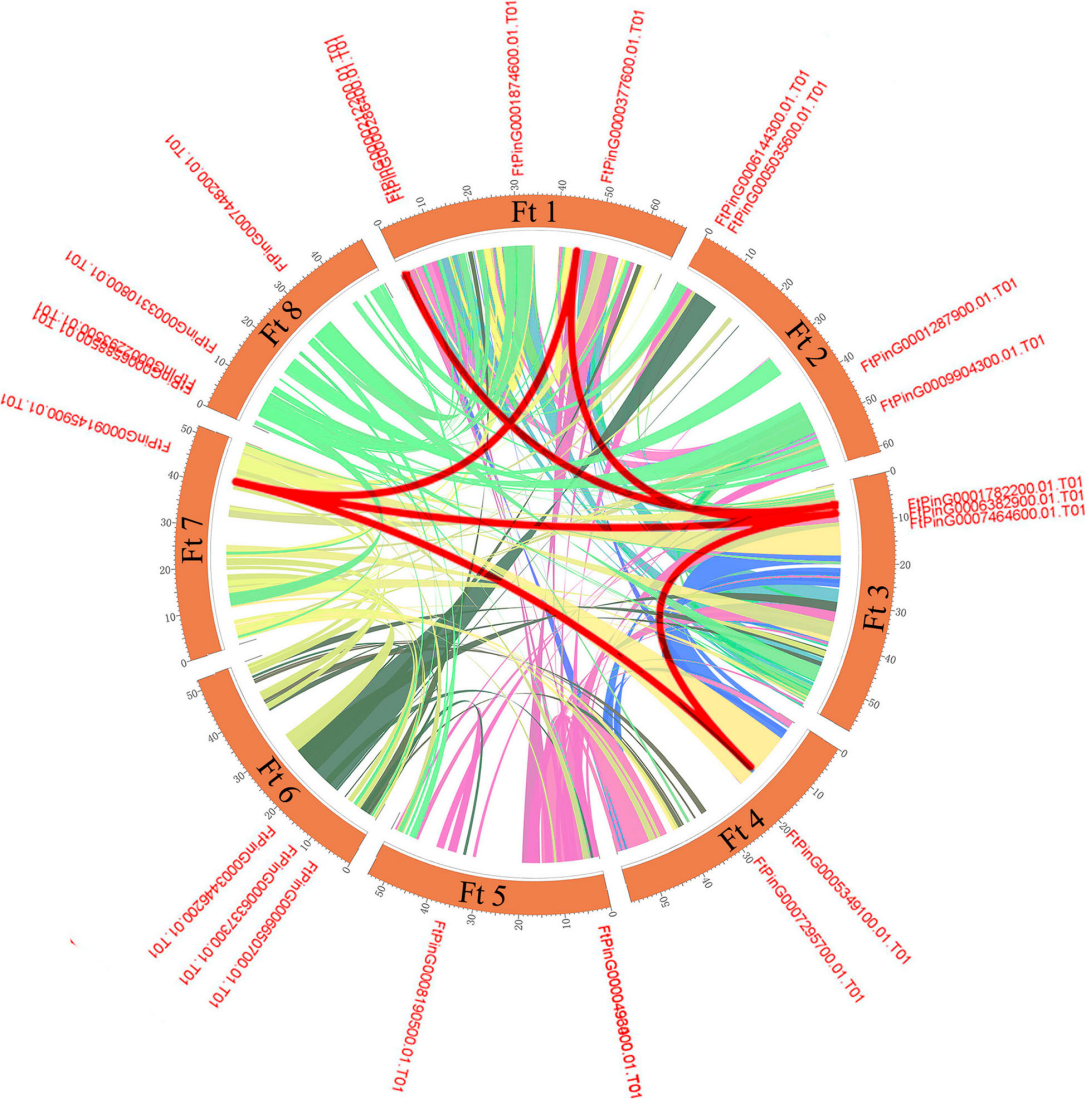
Schematic representations of the interchromosomal relationships of the tartary buckwheat *SPL* genes. Colored lines indicate all syntenic blocks in the tartary buckwheat genome.

Gene duplication events play an indispensable role in the production of new functions and in gene expansion. Therefore, we analyzed the duplication events of the *SPL* genes in the tartary buckwheat genome. There were no tandem duplication events in the tartary buckwheat *SPL* genes, but there were 6 pairs of segmental duplicates (Fig. 4). There are many homologous genes on the different chromosomes of tartary buckwheat, which support the high level of conservation of the SPL gene family. In conclusion, based on the above results, some *FtSPL* genes may have been produced by segmental duplications, and these replication events are the main driving force of *FtSPLs* evolution.

### Evolutionary analysis of the *FtSPL* genes and the *SPL* genes of several different species

The number of *SPL* genes identified in tartary buckwheat was similar to that in *Salvia miltiorrhiza*, apple, maize and Chinese cabbage rice, but the genome size of these five species was very different (tartary buckwheat, 516 Mb; *Salvia miltiorrhiza*, 641 Mb; apple, 742 Mb; maize, 2300 Mb; Chinese cabbage rice, 485 Mb). [21, 23, 25, 28]. This result indicated that the number of SPL family genes was relatively stable in different species during long-term evolution.

**Fig. 5:**
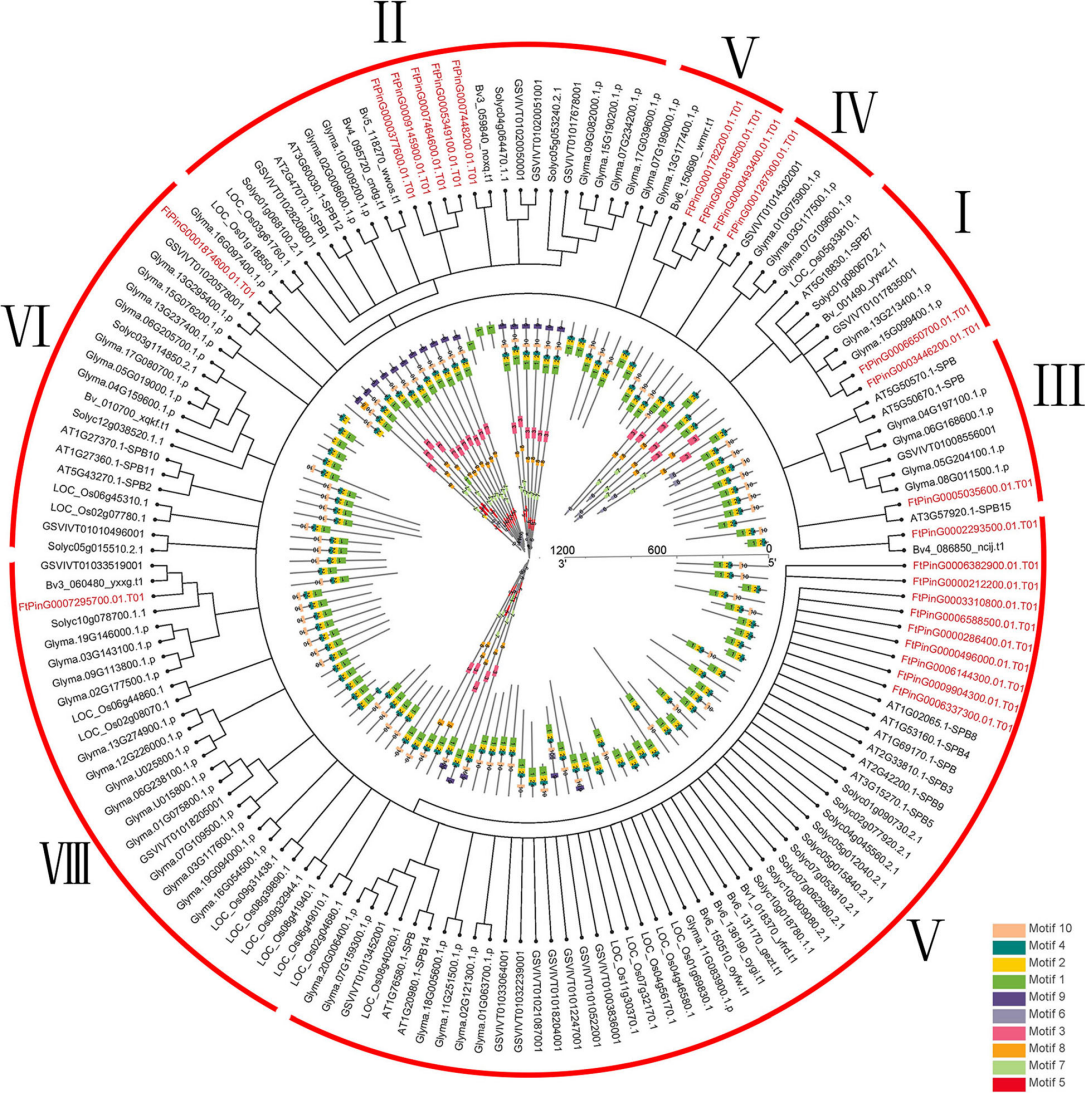
Phylogenetic relationships and motif compositions of the SPL proteins from seven different plant species. Outer layer: An unrooted phylogenetic tree constructed using Geneious R11 with the neighbor-joining method. Inner layer: Distribution of the conserved motifs in SPL proteins. The differently colored boxes represent different motifs and their positions in each SPL protein sequence.

To deduce the evolutionary relationship of the *SPL* genes, a phylogenetic tree analysis was performed for six dicotyledonous plants (*A. thaliana*, *G. max*, *B.vulgaris*, *S. lycopersicum*, *V. vinifera,* and tartary buckwheat) and a monocotyledonous plant *Oryza sativa*. From Fig. 5, we saw that the SPL proteins were divided into 8 groups by the phylogenetic tree, of which group VI had the largest number of members. A total of 10 conserved motifs were detected in the SPL protein sequences of all plants (Additional file 3 Table S2). With the exception of *FtPinG0009145900.01*, *FtPinG0007464600.01* and *FtPinG0005349100.01* only containing motif 1, all other genes contained motif 1, motif 2 and motif 4. All conserved motifs were detected in most *SPL* genes from the other plants in Group II, while several *FtSPL* genes only contained motif 1. Overall, *SPL* genes in the same group of plants had similar motif compositions (Fig. 5).

**Fig. 6:**
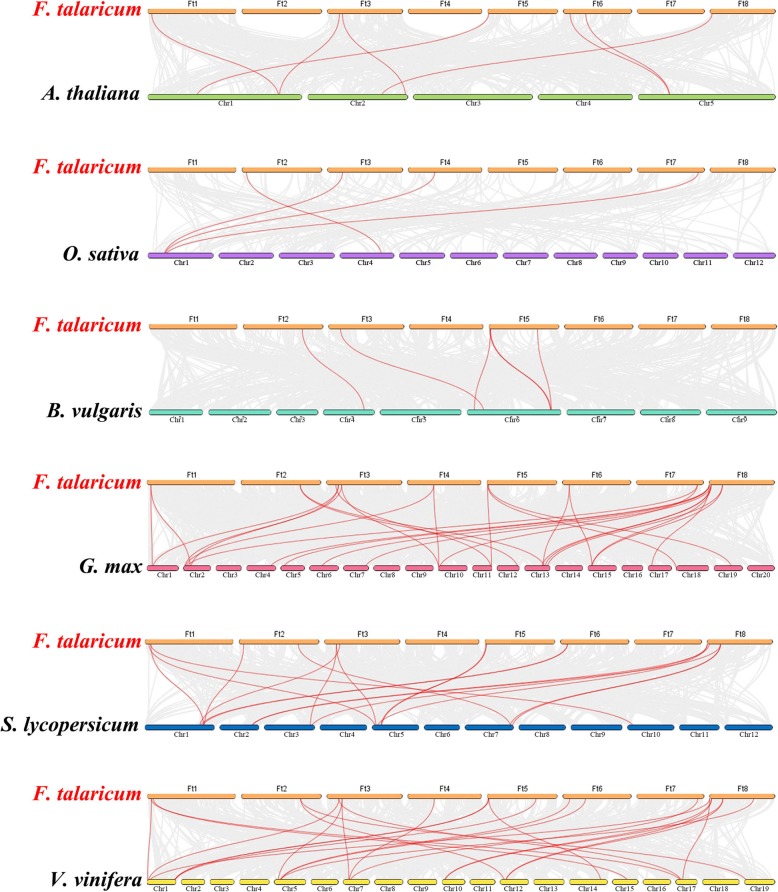
Synteny analyses between the *SPL* genes of tartary buckwheat and six representative plant species. Gray lines in the background indicate collinear blocks within tartary buckwheat and other plant genomes, while red lines highlight syntenic *SPL* gene pairs.

We performed a syntenic analysis of the *SPL* genes in six dicotyledonous plants (*A. thaliana*, *B. vulgaris*, *G. max, S. lycopersicum, V. vinifera* and Tartary buckwheat) and a monocotyledonous plant *O. sativa* to speculate on the evolutionary origin of *SPL* genes. The *SPL* genes in tartary buckwheat were homologous to genes in the reference plants, and the highest level of syntenic conservation was observed among *G. max*, (28 pairs orthologous gene pairs distributed throughout all LGs except LG3, LG8, LG9, LG14, LG16, and LG20), *V. vinifera* (24 orthologous gene pairs distributed on LG1, LG5, LG7, LG10, LG12, LG14, LG15, LG17 and LG19), and *S. lycopersicum* (18 orthologous gene pairs distributed on LG1, LG2, LG3, LG5, LG7 and LG10) (Fig. 6). In the syntenic analysis of the *SPL* genes of tartary buckwheat and *Glycine max*, *FtPinG0006588500.01* was found to be associated with at least three syntenic gene pairs, suggesting that *FtPinG0006588500.01* might play an important role in SPL family evolution (Additional file 4 Table S3). In general, these results indicated that the tartary buckwheat SPL gene family was highly conserved and that the tartary buckwheat *SPL* genes were closer to the *G. max* genes than to *A. thaliana* genes. The *SPL* genes might have evolved from a common ancestor of the different plants.

### Expression patterns of the *FtSPL* genes in different plant tissues

**Fig. 7:**
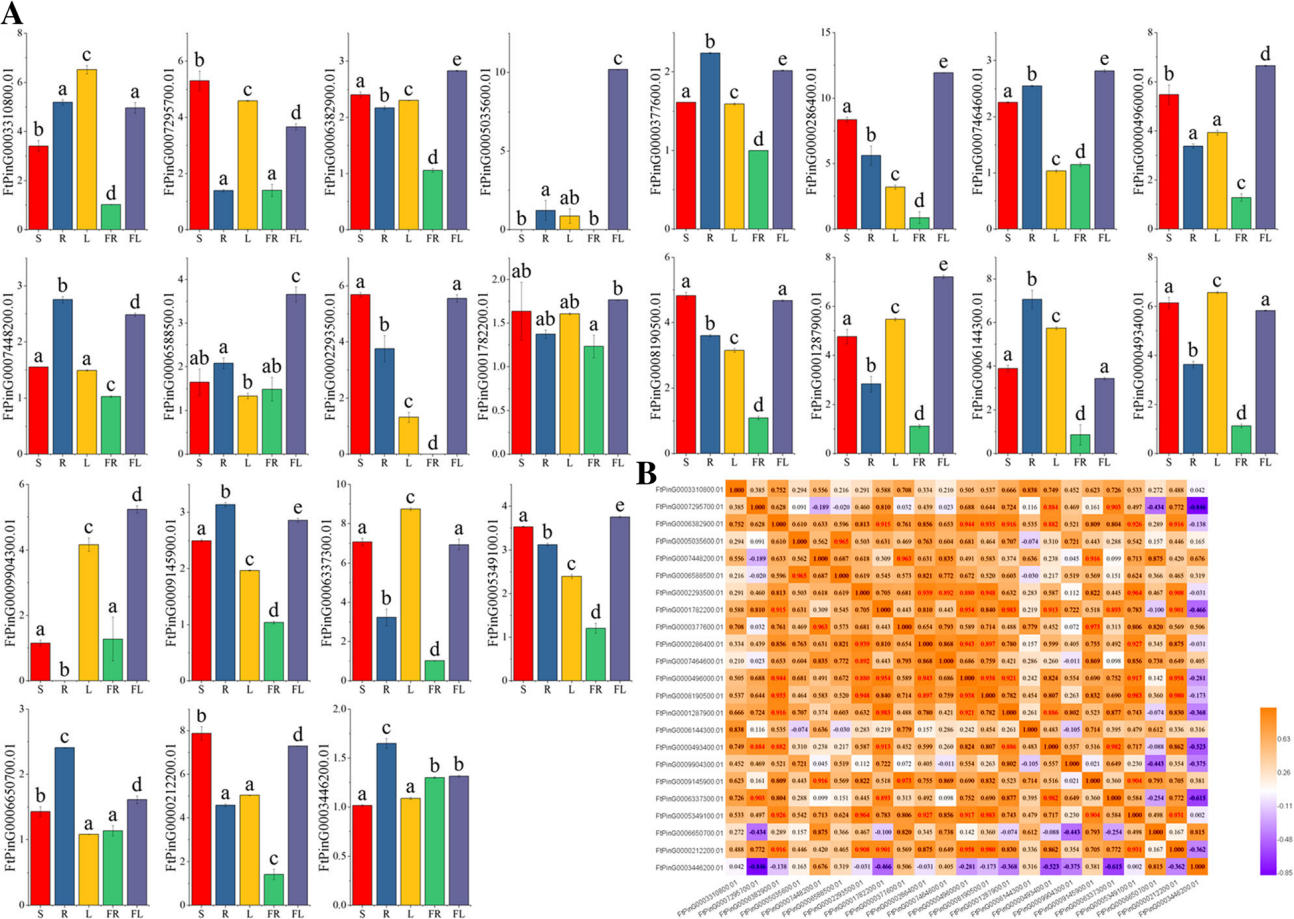
Tissue-specific gene expression of 23 tartary buckwheat *SPL* genes and the correlation between the gene expression patterns of *FtSPLs.***a** The expression patterns of 23 tartary buckwheat *SPL* genes in flower, leaf, root, stem and fruit tissues were examined by qPCR. Error bars were obtained from three measurements. Lowercase letter(s) above the bars indicate significant differences (α = 0.05, LSD) among the treatments. **b** Positive number: positively correlated; negative number: negatively correlated. The red numbers indicate a significant correlation at the 0.05 level.

To further study the physiological functions of the *SPL* genes, we measured the expression patterns of each *SPL* gene in different tissues (roots, stems, leaves, flowers and fruits) of tartary buckwheat by qRT-PCR assay. From the experimental results, we saw that the expression pattern of each gene varied greatly in the different tissues. Among them, 83.3% of the genes (20 genes) were expressed in all tissues, 10 genes (*FtPinG0000286400.01*/*5035600.01*/*1287900.01*/*496000.01*/*9904300.01*/*5349100.01*/*6588500.01*/*6382900.01*/*7464600.01*/*1782200.01*) had the highest expression level in the flowers, and the highest expression levels of 3 genes (*FtPinG0006337300.01*/*3310800.01*/*0493400.01* were found in leaves (Fig. 7). *FtPinG0005035600.01* was not expressed in the stems and fruits, *FtPinG0002293500.01* was not expressed in the fruits, and *FtPinG0009904300.01* was not expressed in the roots. In addition, 10 genes had relatively high expression levels in all tested tissues, including *FtPinG0003310800.1*, *FtPinG0000286400.1*, *FtPinG0000496000.1*, *FtPinG0008190500.1*, *FtPinG0001287900.1*, *FtPinG0006144300.1*, *FtPinG0000493400.1*, *FtPinG0006337300.1*, *FtPinG0005349100.1*, and *FtPinG0000212200.1*. Among all the *FtSPL* genes, *FtPinG0000286400.1* and *FtPinG0006337300.1* showed the highest expression levels in most tissues, with *FtPinG0000286400.1* expressed more highly in the flowers, stems, and roots than in other tissues and with *FtPinG0006337300.1* expressed more highly in the leaves, stems and flowers than in other tissues. *FtPinG0001874600.01* was not expressed in all tissues (not shown in Fig. 7), while the expression levels of *FtPinG0001782200.01* and *FtPinG0003446200.01* were low in all tissues. *FtPinG0001782200.01* was mainly expressed in the flowers and stems, and *FtPinG0003446200.01* was mainly expressed in the roots, flowers and fruits.

In addition, we analyzed the correlations between the expression patterns of the different genes in various tissues. From the correlation analysis, we saw that the expression patterns of most genes were positively correlated; for example, *FtPinG0006382900.01*, which was the mostly expressed in the flowers and had a significant positive correlation with many genes (*FtPinG0000212200.1*/*5349100.01*/*0493400.01*/*1287900.01*/*8190500.01*/*0496000.01*/*1782200.01*) (Fig. 7).

### Differential expression of the *FtSPL* genes during fruit development of tartary buckwheat

Tartary buckwheat fruit is an important functional food. It has a balanced amino acid composition in its proteins with high biological value [4]. Tartary buckwheat fruit also contains higher crude fiber and vitamin B than other fruits, and the content of rutin in tartary buckwheat is also very high [29]. The growth and development of tartary buckwheat fruit may be regulated by some genes, thus affecting the contents of the nutrients in the fruit. Therefore, we can systematically study the expression of the *FtSPL* genes at different stages of fruit development (green fruit stage, discoloration stage and initial maturity stage) and find some genes that potentially regulate fruit growth and development.

**Fig. 8:**
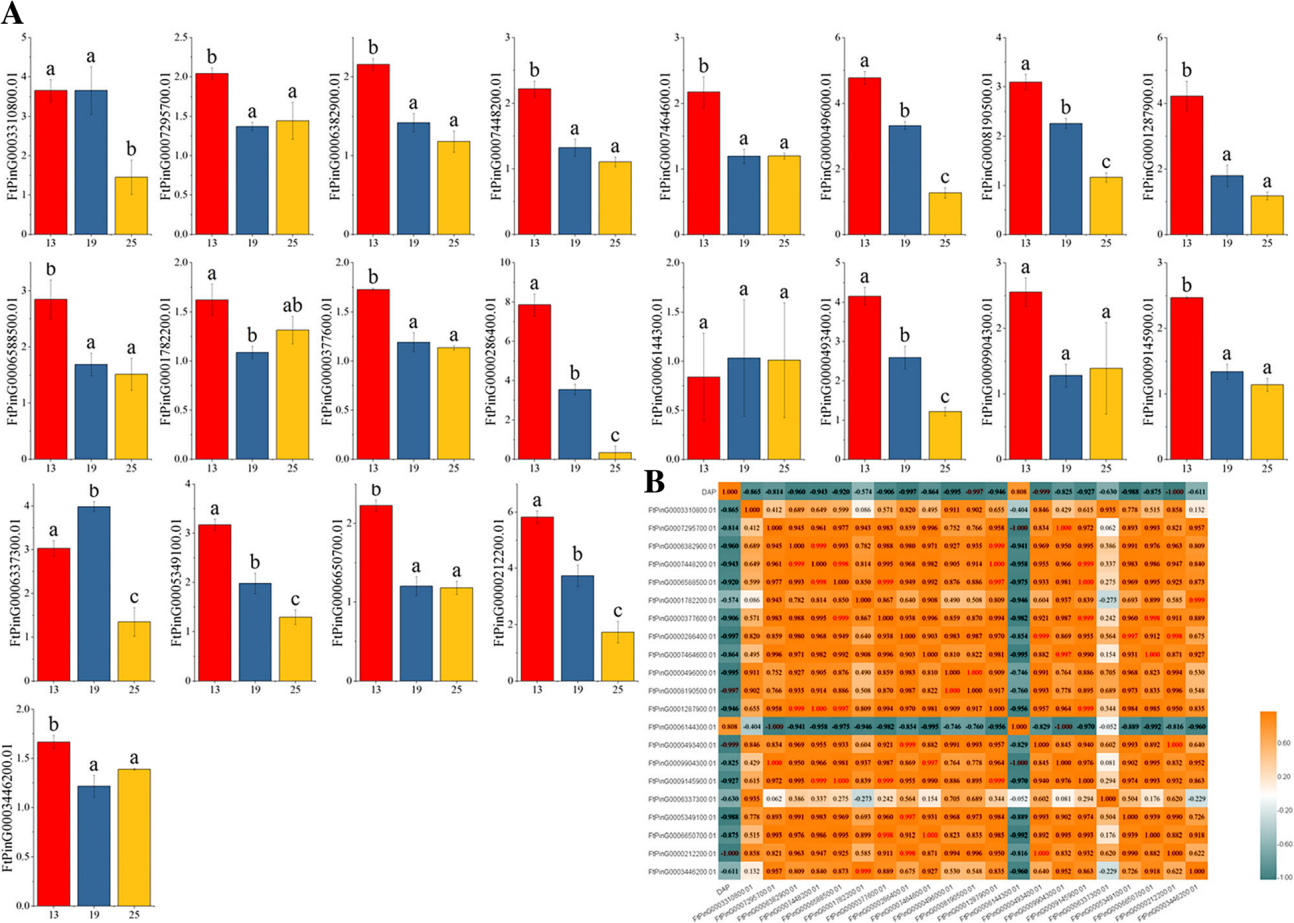
Gene expression of 21 tartary buckwheat *SPL* genes during fruit development and the correlation between the gene expression patterns of *FtSPLs* during fruit development. **a** The expression patterns of 21 tartary buckwheat *SPL* genes in the fruit development stage were examined using a qPCR assay. Error bars were obtained from three measurements. Lowercase letter(s) above the bars indicate significant differences (α = 0.05, LSD) among the treatments. **b** Positive number: positively correlated; negative number: negatively correlated. Red numbers indicate a significant correlation at the 0.05 level

The expression patterns of the *FtSPL* genes at different developmental stages (green fruit stage, discoloration stage and initial maturity stage) were different. Twenty-one genes were expressed at all stages of fruit development. With the development of the fruit, the expression levels of most genes (*FtPinG0006382900.1, FtPinG0007448200.1, FtPinG0000496000.1, FtPinG0008190500.1, FtPinG0001287900.1, FtPinG0006588500.1, FtPinG0000377600.1, FtPinG0000286400.1, FtPinG0000493400.1, FtPinG0009145900.1, FtPinG0005349100.1,* and *FtPinG0000212200.1*) decreased gradually; the expression levels of three genes (*FtPinG0003310800.1, FtPinG0006144300.1* and *FtPinG0006337300.1*) were the highest in the discoloration phase, the expression levels of four genes (*FtPinG0007295700.1, FtPinG0001782200.1, FtPinG0009904300.1* and *FtPinG0003446200.1*) were the lowest in the discoloration stage, and the expression levels of two other genes (*FtPinG0007464600.1* and *FtPinG0006650700.1*) remained stable after the discoloration stage (Fig. 8). Concurrently, we studied the correlations of the *FtSPL* gene expression patterns with tartary buckwheat fruit development and the correlation of each gene during this process. From the results, we saw that there were three genes (*FtPinG0000212200.01/ FtPinG0008190500.01/ FtPinG0000493400.01*) that were significantly negatively correlated with fruit development. Most of the *FtSPL* genes showed a positive correlation. *FtPinG000*6382900.01 was significantly positively correlated with *FtPinG000*1287900.01 and *FtPinG0007448200.01*, while *FtPinG000*7295700.01 was significantly positively correlated with *FtPinG000*9904300.01 but significantly negatively correlated with *FtPinG000*6144300.01 (Fig. 8).

## Discussion

### Evolution of the *SPL* genes in tartary buckwheat

The SPL proteins play an important role in the regulation of plant development, such as in fruit maturation [14, 30], flowering [31] and organ size [32]. The *SPL* gene family has been isolated and identified from many plants, such as *S. lycopersicum* (15 members), *A. thaliana* (17 members), *V. vinifera* (18 members) and *O. sativa* (19 members) [18, 20, 33, 34]. To explore the homology between the *SPL* genes from *A. thaliana* and rice, a phylogenetic tree showed that the *SPL* genes were divided into nine subgroups, while the *SPL* genes in petunia and *A. thaliana* were divided into eight subgroups [33, 35]. The 24 *FtSPLs* identified in this study were also divided into eight subgroups with the genes from *A. thaliana*; each group contained at least one tartary buckwheat *SPL* gene and *A. thaliana SPL* gene (Fig. 1). The number of introns in the *FtSPL* genes ranged from 0 to 10, and the number of introns and motif compositions within each subgroup were similar, which further supports the grouping of the phylogenetic tree (Fig. 2). The *FtSPL* genes were distributed unevenly on 8 chromosomes of tartary buckwheat (Fig. 3). When the homology of the *SPL* genes in the tartary buckwheat genome was analyzed, it was found that there were no tandem duplication gene pairs but 6 pairs of segmental duplications were found (Fig. 4). It is possible that the existence of these homologous genes on the different chromosomes of tartary buckwheat promoted the evolution of the *FtSPL* genes, which allows the number of *SPL* genes in tartary buckwheat to be greater than that in other dicotyledonous plants (*S. lycopersicum*, *A. thaliana*, *V. vinifera* and *O. sativa*).

To further study the evolutionary relationships of the *FtSPL* genes, we constructed a phylogenetic tree consisting of six dicotyledonous plants (tartary buckwheat, *A. thaliana*, *G. max, B. vulgaris, S. lycopersicum* and *V. vinifera*) and a monocotyledonous plant *O. sativa* (Fig. 5). The information we obtained from the phylogenetic tree was that the *SPL* genes from the different plants were also divided into eight groups and that the tartary buckwheat *SPL* genes were scattered among these eight groups. The *FtSPL* genes from group I and group VII were clustered with *SPL* genes in *G. max,* and the *SPL* genes from the other groups were clustered with *SPL* genes in *V. vinifera, B. vulgaris* and *A. thaliana*. A syntenic analysis also showed that the *FtSPLs* had the highest number of homologous gene pairs with *G. max, V. vinifera* and other dicotyledonous plants. When analyzing the motif compositions of the *SPL* genes in many plants, we found that although the motif composition of each subgroup was different, the motif composition within the same group was similar, and almost all the members of group II from all plants contained 10 motifs. These results indicated that the *SPL* genes of tartary buckwheat were closely related to those of the dicotyledonous plants and may have come from the common ancestor.

### Expression patterns and functional prediction of the *FtSPL* genes

At present, there is little functional data on *SPL* genes in tartary buckwheat. Usually, a gene performs a function based on the presence of its expression and to a large extent, the expression patterns of genes are related to the functions of the genes [35]. Transcription factors usually play a key role in controlling the expression of tissue-specific genes [36–38]. In this study, the expression pattern of each *FtSPL* gene was detected by qRT-PCR, and the expression level of each gene was distinct in different tissues (Fig. 7). Most of the *FtSPL* genes were significantly expressed in the flowers, which is similar to the high expression level of *SPL* genes from other species in inflorescences and flower buds [18, 34]. Some genes in the same group showed similar expression profiles; for example, many of the genes in group VI had the highest expression in the flowers (*FtPinG0006382900.01*/*FtPinG0006588500.01*/*FtPinG0000286400.01*/*FtPinG0000496000.01*/*FtPinG0009904300.01*), while genes in group I (*FtPinG0006650700.01*/*FtPinG0003446200.*

*01*) and in group II (*FtPinG0000377600.01*/*FtPinG0007448200.01*/*FtPinG0009145900*) were highly expressed in the roots (Fig. 7). In addition, *FtPinG0006382900.01* in group VI was significantly positively correlated with the genes (*FtPinG0005349100.01*/*FtPinG0000496000.01*) that were highly expressed in the flowers in the same group. In *A. thaliana*, the transcription level of *the SPL15* gene increased during the development process and appeared preferentially in young flowers. Moreover, *SPL15* positively regulated the transformation from the juvenile phase to the adult phase in the vegetative phase of *A. thaliana* and negatively regulated the formation of leaves in *A. thaliana* [39]. *FtPinG0005035600.01* from group III is a homologous gene of *A. thaliana SPL15* (*AtSPL15*), and it can be seen from Fig. 5 that *FtPinG0005035600.01* and *AtSPL15* have similar motifs, *FtPinG0005035600.01* contains motifs 1, 2, 4, and *AtSPL15* contains motifs 1, 2, 4 and 10. Meanwhile, *FtPinG0005035600.01* was expressed only in the flowers, roots and leaves, with very high expression in the flowers and very low expression in the leaves (Fig. 5, Fig. 7). Homologous genes may perform similar functions, so in future studies, we can verify whether *FtPinG0005035600.01* has the same function as *AtSPL15* through further experiments. *AtSPL3* is mainly expressed in inflorescences, and the overexpression of *AtSPL3* not only promotes flowering but also leads to abnormal flower and inflorescence development under long periods of sunlight and continuous illumination [15, 40]. There are many *AtSPL3* homologous genes in tartary buckwheat group VI (*FtPinG0009904300.01/ FtPinG0000496000.01,* etc.*)* and they are highly expressed in flowers. In future research, we can verify whether these genes (*FtPinG0009904300.01/ FtPinG0000496000.01,* etc.*)* have the same function as *AtSPL3* through more in-depth experiments.

Tartary buckwheat has a high nutritional value, and its fruits are rich in rutin, which has antioxidant activity [4, 29]. The growth and development of tartary buckwheat will be regulated by many transcription factors [38, 41–44]. The regulation of the growth and development of tartary buckwheat by transcription factors will directly affect the nutrient content of tartary buckwheat [45, 46]. As the main edible part of tartary buckwheat, fruit growth and development processes are particularly important. Therefore, the expression profile of *FtSPL* genes at different development stages of tartary buckwheat fruits (green fruit stage, discoloration stage and initial maturity stage) can lay a foundation for screening potential genes regulating fruit growth and development. With a qRT-PCR assay, 21 *FtSPL* genes were found to be expressed in different patterns during the fruit development stages (Fig. 8), indicating that the *FtSPL* genes may play different roles in fruit growth and development. *SPL* genes play an important role in the development and maturation of plant fruits, such as *AtSPL10*, which can affect the length of fruit pods, while in rice, *OsSPL16* can control the grain size, shape and quality [14, 47]. In the evolutionary analysis of the *FtSPL* genes, it was found that *FtPinG0007295700.01* and *OsSPL16* (*LOC_OS08g41940.1*) belong to group VII and they have the same motif composition (motifs 1, 2, 4 and 10) (Fig. 5). Previous studies have shown that the green fruit stage is the time at which the fruit size of tartary buckwheat is determined, and the expression level of *FtPinG0007295700.01* was the highest in the green fruit stage (Fig. 8). Therefore, in the future research, we can verify whether *FtPinG0007295700.01* can also regulate fruit size like *OsSPL16* through further more in-depth experiments [48]. *LeSPL-CNR* (*Solyc02g077920.2.1*) is a key gene that controls tomato fruit ripening. Epigenetic variation in the promoter region of *LeSPL-CNR* affects the biosynthesis of carotenoids and cell wall structure, which inhibits the normal ripening of fruit [30, 49]. *LeSPL-CNR* and tartary buckwheat *SPL* genes were clustered in group VI, in which *FtPinG00063829.00.01, FtPinG0000212200.01, FtPinG0006588500.01 and FtPinG0000286400.01* had the same motif compositions as *LeSPL-CNR*, and these *FtSPL* genes were also highly expressed in the flowers (Fig. 5, Fig. 7). Based on these experimental results, the specific functions of these *FtSPL* genes can be verified by further experiments in the future.

## Conclusion

These results are the first genome-wide analysis of the *SPL* gene family in tartary buckwheat.

We comprehensively analyzed 24 putative *FtSPL* genes, including their gene structures, conserved motifs, gene duplication, evolutionary relationships and spatial and temporal expression patterns, which may be related to their biological functions**.** The phylogenetic tree divides the *FtSPL* gene into eight groups, each of which has similar motif compositions and gene structures. The expression patterns of these *FtSPL* genes in different tissues suggest that these *FtSPL* genes may regulate the growth and development of tartary buckwheat. Our research lays a foundation for the future elaboration of the potential functions of the *FtSPL* genes in the growth and development of tartary buckwheat.

## Methods

### Plant material

The tartary buckwheat accessions (XIQIAO) used in this study were requested from Professor Wang Anhu of Xichang University. The materials were planted in the experimental field of the College of Life Science, Sichuan Agricultural University (Lat. 29°97′ N, 102°97′ E, Alt. 580 m), Ya’an, Sichuan, China. The samples including flowers, fruits from three (13, 19, and 25 days after pollination, DAP) different developmental fruit stages, and the stems, roots, and leaves of mature tartary buckwheat were collected separately, quickly put into liquid nitrogen and stored at − 80 °C for further use.

### Identification of the *SPL* genes in tartary buckwheat

The tartary buckwheat genome was downloaded from the Tartary Buckwheat Genome Project (TBGP; http://www.mbkbase.org/Pinku1/). The largest number of *SPL* genes was screened from the tartary buckwheat genome by two BLASTp methods, and the Hidden Markov Model (HMM) profiles corresponding to the SBP domain *(PF03110)* were downloaded from the Pfam protein family database (http://pfam.xfam.org/). The detailed method is as follows: we downloaded the amino acid sequences of all SPL proteins in *A. thaliana* from tair library, which range from 131aa to 1035aa. We identified all similar *SPL* genes from tartary buckwheat genome by using *SPL* gene sequences in *A. thaliana* as a target. After that, we analyzed the conserved domain of *FtSPL* genes, removed the gene that did not contain SBP conserved domain, and finally 24 genes containing the SBP domain were screened from the tartary buckwheat genome. The PIs, MVs and subcellular localizations of the *FtSPL* genes were analyzed on the ExPASy website (https://web.expasy.org/compute_pi/).

### Phylogenetic analyses and intron-exon structure determination

The SPL protein sequences (*A. thaliana*, *B. vulgaris*, *G. max*, *S. lycopersicum*, *V. vinifera* and *O. sativa*) for N-J phylogenetic trees were downloaded from the UniProt database (https:/www.uniprot.org). Multiple amino acid sequences of identified *SPL* genes were aligned using Clustalx1.81 program. The phylogenetic trees comparing tartary buckwheat and multiple species (*A. thaliana*, *B. vulgaris*, *G. max*, *S. lycopersicum*, *V. vinifera* and *O. sativa*) were constructed with the NJ method and the specific parameters were Poisson model and 1000 bootstrap replications. The phylogenetic trees comparing tartary buckwheat and *A. thaliana* were also constructed with Mega 7.0 by the N-J method and parameters above. SPL protein sequences from tartary buckwheat and *A. thaliana* were also aligned using Clustalx1.81 program before the phylogenetic tree was constructed. In addition, the online Gene Structure Display Service (http://gsds.cbi.pku.edu.cn) was used to predict the intron structure by comparing the cDNA of the *FtSPL* genes with the corresponding genomic DNA sequences. The determination of the conserved motifs in FtSPL proteins was conducted by the MEME online program (http:/meme.nbcr.net/meme/intro.html), and the parameters were set to the optimum mode width of 6 to 200 and the maximum number of motifs of 10.

### Chromosomal distribution and gene duplication of the *FtSPL* genes

The method of mapping *FtSPL* genes to the chromosomes of tartary buckwheat was performed according to Liu et al. [50]. Analysis of the *FtSPL* gene replication events was conducted using multiple collinear scanning toolkits (MCScanX). The syntenic relationship between the *FtSPL* genes and *SPL* genes from selected plants was determined by using Dual Synteny Plotter software (https://github.com/CJ-Chen/TBtools).

### Expression analysis of the *FtAP2/ERF* genes by real-time PCR

The qRT-PCR primers were designed with primer 3 software (http://frodo.wi.mit.edu/) (Additional file 5 Table S4). The design criteria of primers were as follows: PCR product length range of 100 to 200, GC content is 50–60% and primer melting temperatures range of 62 °C to 67 °C were selected. Using the tartary buckwheat histone H3 gene as an internal reference gene, SYBR Premix Ex Taq II (TaKaRa) was used to carry out qRT-PCR [50]. Each experiment was repeated at least three times, and the data were analyzed by the 2^−(∆∆Ct)^ method [51].

### Statistical analysis

All the data were analyzed by analysis of variance using the Origin Pro 2018b (OriginLab Corporation., Northampton, Massachusetts, USA) statistics program, and the means were compared by the least significant difference test (LSD) at significance levels of 0.05 and 0.01.

## Additional files

Additional file 1: **Figure S1.** Alignment of multiple *FtSPL* and select SBP domain amino acid sequences. (DOCX 799 kb)

Additional file 2: **Table S1.** List of the 134 FtAP2/ERF genes identified in this study. (XLS 114 kb)

Additional file 3: **Table S2.** Analysis and distribution of the conserved motifs in tartary buckwheat AP2/ERF proteins. (XLS 36 kb)

Additional file 4: **Table S3.** One-to-one orthologous relationships between tartary buckwheat and other plants. (XLS 72 kb)

Additional file 5: **Table S4.** The primer sequences for qRT-PCR. (XLS 33 kb)

## Data Availability

The genome sequences of tartary buckwheat used for identifying the *AP2/ERF* genes in this study were located in the Tartary Buckwheat Genome Project (TBGP; http://www.mbkbase.org/Pinku1/). The tartary buckwheat accession (XIQIAO) materials used in the experiment were supplied by Professor Wang Anhu of Xichang University. The datasets supporting the conclusions of this article are included in the article and its Additional files.

## Abbreviations

*A. thaliana*: *Arabidopsis thaliana*
ABA: Abscisic acid
*B. vulgaris*: *Beta vulgaris*
*C. annuum*: *Capsicum annuum* L
CDS: Coding sequence
DAP: Days after pollination
Ft: *Fagopyrum tataricum*
FtSPL: *Fagopyrum tataricum* SPL
*G. max*: *Glycine max*
HMM: Hidden Markov Model
LG: Linkage group
LSD: least significant difference
*M. domestica*: *Malus domestica* Borkh
MVs: Molecular weights
NLS: Localization signal
*O. sativa*: *Oryza sativa*
PIs: Isoelectric points
qRT-PCR: Quantitative real-time polymerase chain reaction
*R. communis* L: *Ricinus communis* L
*S. lycopersicum*: *Solanum lycopersicum*
S. miltiorrhiza: Salvia miltiorrhiza
SQ-UA: SQUAMOSA
TBGP: Tartary buckwheat genome project
TFs: Transcription factors
*V. vinifera*: *Vitis vinifera*
*Z. mays*: *Zea mays* L
