# Supplementary material for: Genome-wide identification of the SPL gene family in Tartary Buckwheat (Fagopyrum tataricum) and expression analysis during fruit development stages
Source: BMC Plant Biol. 2019 Jul 8;19:299. doi: 10.1186/s12870-019-1916-6 (PMC6615263; doi:10.1186/s12870-019-1916-6)
Supplement: Supplementary file 1 — Figure S1. Alignment of multiple FtSPL and select SBP domain amino acid sequences. (DOCX 799 kb) [file 12870_2019_1916_MOESM1_ESM.docx]

**Title:** Genome-wide identification of the *SPL* gene family in Tartary Buckwheat (*Fagopyrum tataricum*) and expression analysis during fruit development stages

**Running Title:** SPL proteins in tartary buckwheat fruit

Moyang Liu^1, †^, Wenjun Sun^1, †^, Zhaotang Ma^1^, Tianrun Zheng^1^, Li Huang^1^, Qi Wu^1^, Zizhong Tang^1^, Tongliang Bu^1^, Chenglei Li^1^, and Hui Chen^1, *^

^1^*Sichuan Agricultural University, College of Life Science, Ya’an, China*

^†^These authors contributed equally to this work.

**^*^Corresponding authors:** Hui Chen, email: chenhui@sicau.edu.cn, phone (+86) 18981604486

**Figure S1** Alignment of multiple *FtSPL* and select SBP domain amino acid sequences.**
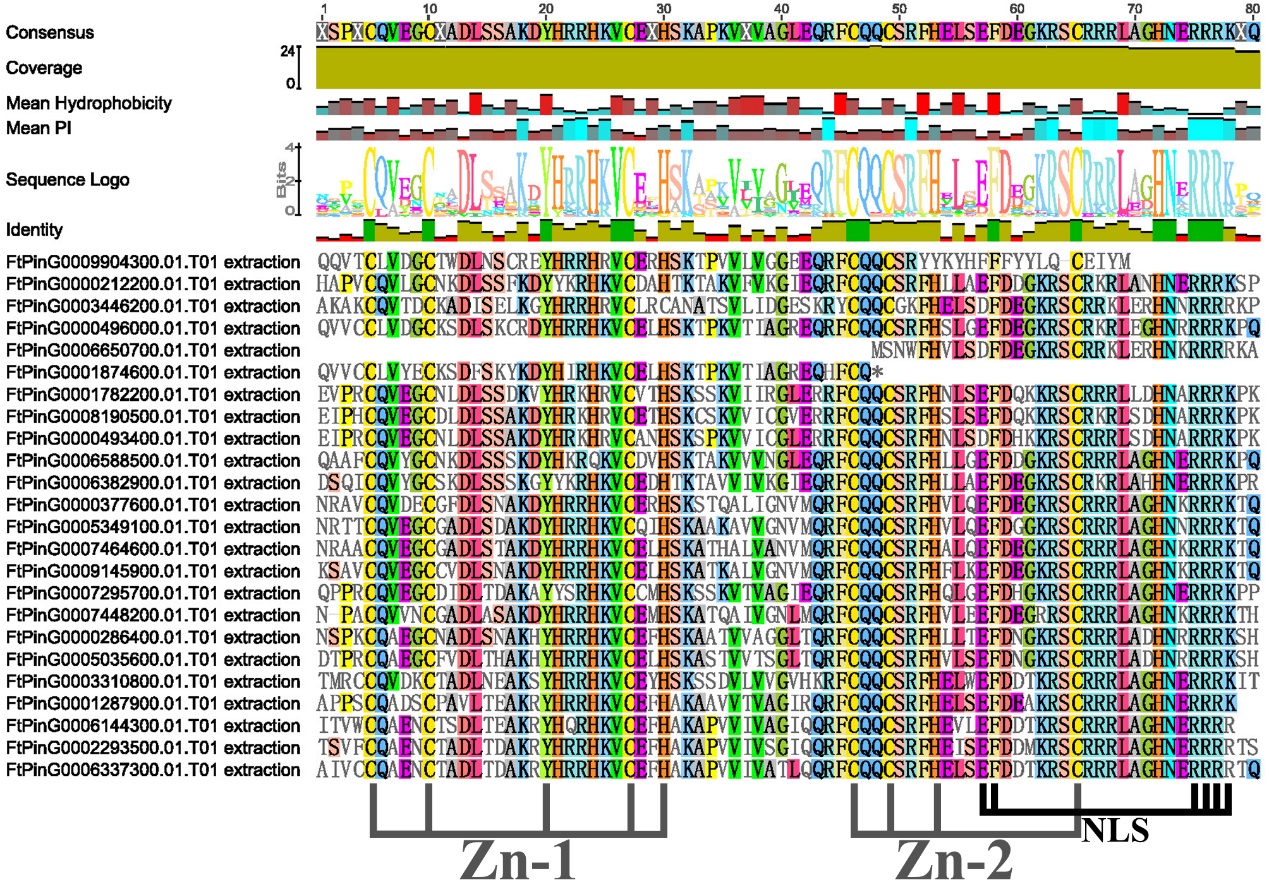
**
